# Sensors for Ultrasonic Nondestructive Testing (NDT) in Harsh Environments

**DOI:** 10.3390/s20020456

**Published:** 2020-01-14

**Authors:** Anthony N. Sinclair, Robert Malkin

**Affiliations:** 1 Department of Mechanical and Industrial Engineering, University of Toronto, 5 King’s College Road, Toronto, ON M5S 3G8, Canada; 2 Department of Mechanical Engineering, University of Bristol, Bristol BS8 1TR, UK; Rob.Malkin@bristol.ac.uk

**Keywords:** ultrasound, nondestructive testing, ultrasonic transducer, high temperature, radiation

## Abstract

In this special issue of *Sensors*, seven peer-reviewed manuscripts appear on the topic of ultrasonic transducer design and operation in harsh environments: elevated temperature, high gamma and neutron fields, or the presence of chemically aggressive species. Motivations for these research and development projects are strongly focused on nuclear power plant inspections (particularly liquid-sodium cooled reactors), and nondestructive testing of high-temperature piping installations. It is anticipated that we may eventually see extensive use of permanently mounted robust transducers for in-service monitoring of petrochemical plants and power generations stations; quality control in manufacturing plants; and primary and secondary process monitoring in the fabrication of engineering materials.

## 1. Introduction

Industrial use of ultrasound for nondestructive testing (NDT) has expanded rapidly over recent years, but has generally been confined to inspections at ambient temperatures, in radiation-free, chemically-benign surroundings. Most of the sensors are based on a lead-zirconate-titanate (PZT) piezoelectric element that has a Curie temperature of a few hundred degrees Celsius, and various plastic components that would not survive temperature excursions above 200 °C. In addition, the degradation of adhesives, and breakdown of various transducer components would threaten such traditional ultrasonic transducers exposed to harsh environments.

In the face of increasingly stringent demands from regulatory bodies that oversee critical industries, and demands from industrial leaders, the demand for ultrasonic transducers that can withstand harsh conditions has risen sharply. Key drivers include:

- On-line, full-power monitoring of crack growth, corrosion, and flow rates in hot piping systems (e.g., nuclear power plants and petrochemical installations).
- Process control and immediate inspection of products on manufacturing production lines (e.g., steel mills and casting plants).
- Nuclear power accident investigation and monitoring as well as nuclear decommissioning inspection/assessment of contaminated materials and structures.
- Corrosion monitoring in chemically aggressive environments (e.g., geothermal piping systems).

There are various mechanisms that have been explored to deal with harsh environments for ultrasonic sensors. These include a selection of materials that resist degradation; use of forced (water) cooling systems; delay lines and wedges that effectively isolate the transducer from the aggressive agent; and air-coupling or electromagnetic acoustic transducers (EMATs) that place a layer of air between the sensor and item under inspection. In this special issue of *Sensors*, seven peer-reviewed manuscripts explore some of the engineering challenges encountered with the design and operation of ultrasonic transducers under these imperfect conditions.

## 2. Contributions

The first paper by Dhutti et al. [1] from Brunel University UK, focuses on the on-line integrity monitoring of high temperature piping systems, by the use of a piezoelectric wafer active sensor to generate torsional guided waves. As piping temperatures reach up to 600 °C, a gallium phosphate (GaPO_4_) piezoelectric element was selected, which will resist depoling over extended time periods. Challenges such as differential thermal expansion of transducer components, characterization of material properties, and generation of multiple modes are explored with the aid of a finite element model. Testing conducted for periods of over 1000 h indicate the potential for permanently mounted transducers on piping systems for long term condition monitoring.

The second paper by Bhadwal et al. [2] investigates the problem of coupling together the various components of an ultrasonic transducer that would undergo excursions to temperatures as high as 700 °C. Due to the differential thermal expansion of the piezoelement, backing layer, and wear plate, solid coupling techniques such as brazing would lead to cracking or even total transducer failure. The authors evaluated the feasibility of using dry coupling, but at far lower clamping loads than the 200–500 MPA interfacial pressure often quoted for high dry coupling efficiency. Their transducer design is spring-loaded to maintain a constant inter-layer pressure as the transducer undergoes large temperature excursions; using soft coupling foils at each transducer component interface demonstrated that dry coupling at pressures of 25 MPA can lead to a high signal-to-noise ratio (SNR) and signal amplitude.

The third paper by Pucci et al. [3] originates from the French Alternative Energies and Nuclear energy Commission (CEA) which has a strong program for development of liquid sodium-cooled fast reactors. The ultrasonic sensor is a 12-element array based on the principle of an EMAT array designed for in-service reactor inspection. The paper is concentrated on design details and laboratory testing of prototype transducers; CIVA simulation software is used for simulation and to optimize focusing characteristics. A major advantage of EMAT design is that very accurate control of beam steering and mode generation can be achieved through a judicious choice of array architecture, coil geometry, and magnet arrangement. The inherently weak transduction mechanism of an EMAT is a major challenge, addressed to the extent possible in the design process.

The fourth paper (also from the French CEA) by Le Jeune et al. [4] is again motivated by interest in ultrasonic imaging in in-service sodium-cooled fast reactors. However, the focus here is on optimization of a long-distance imaging system that consists of two linear phased arrays (termed antennas) that are perpendicular to each other. Full Matrix Capture is used for data collection, and then Total Focusing Method is applied to the data for imaging of large areas. CIVA simulations of the system are compared with experimental results of data collected in water; a prototype system for under-sodium trials is currently being manufactured. Extensive plans are described to reduce computation time and improve SNR.

The fifth paper by Saillant et al. [5] also deals with the design of a transducer to be used for inspections conducted in liquid sodium, at temperatures on the order of 200 °C. Although the proposed transducer is based on the same principles of conventional room-temperature probes, the combination of elevated temperature, radiation fields, and a chemically aggressive environment necessitates several design modifications and a selection of appropriate materials. A significant addition is a “wetting layer” to allow ultrasound to pass efficiently between the transducer and liquid sodium. Extensive test result are presented for inspection of machined defects in a welded stainless steel test block.

The sixth paper by Tittmann et al. [6] originates from a long record of research at Penn State University into ultrasonic transducers for use in nuclear reactors. A very thorough review is provided of the piezoelectric materials that could be used under conditions of temperatures over 1000 °C, neutron fluence up to 10^20^ cm^−2^, and gamma ray dose rates of up to 10^9^ Rem/h. Alternative configurations include spray-on transducers and guided wave sensors. Experimental results are presented of transducer performance as a function of irradiation time (both gamma and neutron irradiation) and temperature, for a variety of piezomaterials. This review includes a very extensive bibliography of work done on characterization of piezomaterial properties, and transducer designs for harsh environments.

The last paper of this special issue of *Sensors* comes from the Korean University of Science and technology. Geonwoo Kim et al. [7] are developing an ultrasonic transducer for a very specific task: inspection of pressurized water reactors fuel rods for the detection of infiltrated water (akin to fuel rod failure). A high-sensitivity Pb(Mg_1/3_Nb_2/3_)O_3_-PbTiO_3_ single crystal is selected as the active piezoelement, as it can generate stronger signals than PZT-based transducers, in this highly attenuative inspection environment. Extensive results are reported on the effect of neutron irradiation on this material, to show that it is suitable for an in-reactor environment. Transducer design details, backed up by a one-dimensional Krimholtz Leedom Matthae (KLM) model of performance, are presented, for a sensor with central frequency of 5 MHz.
